# Pseudogout Associated Hip Pain in a Patient with HIV Infection

**DOI:** 10.1155/2010/842814

**Published:** 2010-12-20

**Authors:** Benan M. Dala-Ali, Matthew Welck, Mary Anne Lloyd, Henry D. Atkinson

**Affiliations:** ^1^North Middlesex University Hospital, Sterling Way, London N18 1QX, UK; ^2^Watford General Hospital, Hertfordshire WD18 0HB, UK

## Abstract

HIV infection is a global pandemic, currently affecting approximately 77,000 people in the UK and 33 million people around the world. The infection has widespread effects on the body and can involve the musculoskeletal system. It is therefore important that orthopaedic surgeons are aware of the condition and its sequelae. We present the case of a 46-year-old man with a 10-year history of HIV who presented with acute hip pain, difficulty weight-bearing, and constitutional symptoms. Following radiological, microbiological, and serological tests a diagnosis of pseudogout was established following microscopic analysis of the hip joint aspirate. The patient's symptoms resolved completely following the joint aspiration and NSAID therapy. Studies have shown a relationship between HIV infection and gout. The virus has also been linked to osteonecrosis, osteopenia, bone and joint tuberculosis, and septic arthritis from rare pathogens. However, it is difficult to fully ascertain whether these conditions are related to the HIV infection itself or the HAART (highly active antiretroviral therapy). There are no previously reported cases of HIV-infected patients with pseudogout. The case is discussed with reference to the literature.

## 1. Background

HIV infection is a global pandemic which in 2007 affected 77,000 people in the UK and 33 million people around the world, according to the World Health Organisation [1]. The infection has widespread systemic effects which include the musculoskeletal system. It is thus essential that orthopaedic surgeons are aware of the condition and its sequelae.

Antiretroviral therapies, first licensed in 1995, have altered the course of HIV and its manifestations. However, these drugs are also known to have a multitude of side effects including osteonecrosis and metabolic abnormalities. 

We report the case of an HIV patient presenting with spontaneous non-traumatic hip pain, who following radiological, microbiological, and serological tests was diagnosed as suffering from pseudogout. This is the first reported case of an HIV-infected patient with hip pseudogout. The case is discussed in the context of HIV and other causes of acute joint pain.

## 2. Case Report

A 55-year-old HIV-positive Caucasian man presented with a one-day history of spontaneous-onset left hip pain. The patient described a similar episode in the contralateral hip in 1997, which had resolved with analgesia and rest.

He had been diagnosed with HIV in 2002 and was immediately commenced on “highly active antiretroviral therapy” (HAART) to control disease progression. His HIV treatment consisted of two nucleoside reverse transcriptase inhibitors (emtricitabine and tenofovir) given as a fixed-dose combination (Truvada 200/245) once per day and the protease inhibitor duranavir 800 mg boosted with ritonavir 100 mg once per day. The patient also had a history of hypercholesterolaemia and syphilis. He was a non-smoker and an infrequent drinker.

On presentation he was mildly pyrexial and was unable to weight-bear through pain. There was a reduced range of both active and passive left hip movement (20–75 degrees flexion and 10-0-10 degrees of rotation), and it was held in 30 degrees of flexion at rest. Other musculoskeletal and neurological examinations were essentially normal. Blood investigations initially found a normal white blood cell count (WCC) of 8.3 × 10^9^/L, C-reactive protein (CRP) of <3 U/L, erythrocyte sedimentation rate (ESR) of 10 mm/hr, Creatine Kinase (CK) of 88 U/L (normal 25–150 U/L), and negative ANA and ANCA. 

**Figure 1:**
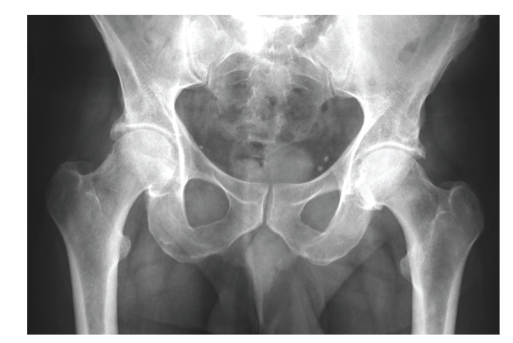
X-ray at admission.

Plain radiographs of the pelvis demonstrated only mild bilateral osteoarthritis (see Figure 1).

**Figure 2:**
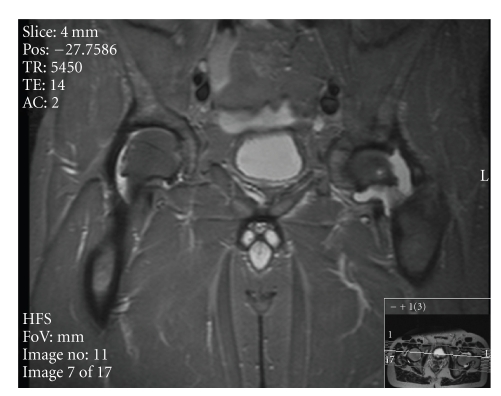
T2 weighted MRI scan of the pelvis.

The patient was admitted under the HIV physicians for bed rest, analgesia, and observation; however he did not improve. Over the subsequent two days his CRP increased to 109, ESR to 90, though his white cells remained stable (WCC of 8.1 × 10^9^/L). An MRI scan revealed a large left hip effusion and a moderate right hip effusion (see Figure 2). 

An aspiration of the left hip was performed under local anaesthetic and revealed a turbid yellow fluid, which was sent for microbiology and cytology. A differential count of the aspirate showed 95% neutrophils, and gram staining found no bacteria present. Microscopy revealed positively birefringent crystals, consistent with calcium pyrophosphate. A diagnosis of hip pseudogout was made and the patient was commenced on high-dose nonsteroidal anti-inflammatory drugs (NSAIDs). The patient improved clinically over the next 48 hours and was discharged home fully weight-bearing. He was followed up in both the orthopaedic and rheumatology clinics, where the latter excluded any other major risk factors for the development of pseudogout. He remained well at 6-month review.

## 3. Discussion

HIV has been linked with a variety of orthopaedic and rheumatological conditions. The first documented association in 1987 was between AIDS and Reiter's Syndrome, followed by gout, osteoporosis, avascular necrosis, septic arthritis, osteomyelitis, and tuberculosis [2]. 

30%–40% of HIV/AIDS patients suffer from arthralgia. Though this can affect any joint, the knees, shoulders, and elbows are most commonly affected. When presented with such a patient, the orthopaedic surgeon must be aware of the possible differential diagnoses.

### 3.1. Gout

Gout is estimated to affect around 0.5% of HIV/AIDS patients per year [3, 4]. These patients often have urate abnormalities, 41% with hyperuricaemia and 5% with hypouricaemia, unlike HIV-negative patients whose urate levels are usually normal [5]. The elevated urate is likely to be the result of the HAART treatment itself [6–8] rather than of the HIV infection, and drugs such as the protease inhibitor ritonavir are known to have this association [9]. There are several hypotheses for why HAART might cause hyperuricaemia. The drugs may cause mitochondrial toxicity, which can increase the formation of lactate, which then competes with urate for tubular secretion in the kidneys. HAART drugs may also cause a respiratory chain failure that results in ATP depletion, which then increases urate production in the purine nucleotide cycle [10, 11]. Thirdly, HAART drugs cause hyperlipidaemia, insulin resistance, and central adiposity, which in turn can lead to gout.

### 3.2. Pseudogout

Despite the association between gout and HIV, there have been no documented cases of pseudogout-associated arthralgia. Whilst most cases of pseudogout are idiopathic, there are relationships with trauma, aging, and metabolic diseases, including hyperparathyroidism and haemochromatosis. Calcium pyrophosphate crystals in pseudogout are thought to develop from an increased adenosine triphosphate breakdown, which can lead to increased pyrophosphate levels in the joint [12]. 

It is difficult to know whether the HIV infection or HAART therapy was directly responsible for the pseudogout or whether it was secondary to another condition. Studies have shown a link between HIV or HAART and hypophosphatemia [13], hyperparathyroidism [14], hypothyroidism [15], and hypercalcaemia [16]. All these conditions are known risk factors of pseudogout.

### 3.3. Septic Arthritis

Septic arthritis usually presents with a short history of joint pain and fever, with a raised ESR and an absence of peripheral leukocytosis. It is more prevalent in HIV/AIDS patients that are intravenous drug users or haemophiliacs [17, 18], and the majority resolve with appropriate intravenous antibiotics [5]. However, if the patient fails to improve on the antibiotics, an open drainage of the joint should be performed in theatre. The most common pathogens are *Staphylococcus aureus* (60%) and *Candida albicans* (20%), and rarely pathogens such as *Stenotrophomonas maltophilia* and *Proteus wickerhamii* [19].

### 3.4. Tuberculosis

There has been a dual global epidemic of tuberculosis (TB) and HIV/AIDS. The World Health Organisation estimated that there were 9.27 million new cases of TB in 2007 (139 per 100,000 population); of these 1.37 million (14.8%) were HIV positive. The two conditions are interlinked, as the HIV-virus specifically eliminates macrophages and CD4 lymphocytes, which are cells essential for the prevention of active tuberculosis. 

There has been an increased incidence of extrapulmonary TB, including bone and joint TB, in HIV/AIDS patients [20]. The diagnosis of skeletal TB is often delayed in developed countries, as it is not commonly encountered. 

The most common site of musculoskeletal TB among HIV/AIDS patients is the spine (Pott's spine) [5]. The infection can also affect the weight-bearing joints, in particular the knee and hip. It presents with chronic pain with minimal inflammation. TB osteomyelitis classically presents with pain and swelling of the bone and surrounding soft tissues. There may also be enlarged regional lymphadenopathy or the presence of an abscess or sinus.

### 3.5. Osteopenia/Osteoporosis

HAART causes metabolic changes in the body and has been shown to cause osteopenia and osteoporosis, putting patients at risk of low-energy fractures. A study of 600 HIV-infected individuals on antiretroviral therapy demonstrated a significantly higher prevalence of osteopenia compared to the national average in the US [21].

The underlying mechanism of bone loss in HIV-infected patients is not fully understood. Studies using markers of bone formation and resorption have shown an uncoupling of these events in individuals with HIV infection [22]. Other studies have found an increased number of proinflammatory cytokines tumour necrosis factor (TNF) and interleukin-6 (IL-6) in the HIV-infected patients, which have an important role in osteoclast activation and resorption [23].

### 3.6. Osteonecrosis

HIV/AIDs patients have an increased risk of developing osteonecrosis, believed to occur as a result of a vascular thrombosis, caused either by the actions of anticardiolipin antibodies or by a deficiency of protein S [24]. A study using magnetic resonance imaging (MRI) of 339 HIV-positive patients found that 4.4% suffered from asymptomatic osteonecrosis [25]. It is unclear whether this is the result of the HIV virus, the HAART treatment, or an increased appreciation of osteonecrosis on scans. The protease inhibitor drugs have been specifically reported to lead to osteonecrosis [26, 27].

## 4. Conclusion

HIV/AIDS is a global phenomenon and it is essential that clinicians are aware of its widespread effects and those of its drug treatments. This is the first documented case of hip pseudogout in an HIV/AIDS patient. Though it is unclear whether this was the result of the HIV infection, the HAART treatment, or an unrelated cause, the cases highlight the diagnostic difficulties one can encounter in these susceptible patients.

##  Conflict of Interests

There is no conflict of interests. The authors received no financial or other type of support to carry out this study. This is an original paper and has not been published in any other journal. All authors read and approved this paper for publishing purposes.

##  Consent

Written informed consent was obtained from the patient for publication of this case report and any accompanying images.

##  Authors' Contributions

B. M. Dala-Ali, M. Welck, and H. D. Atkinson managed the patient. B. M. Dala-Ali and M. A. Lloyd wrote the paper. M. Welck and H. D. Atkinson assisted with the literature review and paper preparation. All authors have read and approved the final paper.
